# Nemo-Like Kinase (NLK) Is a Pathological Signaling Effector in the Mouse Heart

**DOI:** 10.1371/journal.pone.0164897

**Published:** 2016-10-20

**Authors:** Ruijie Liu, Hadi Khalil, Suh-Chin J. Lin, Michelle A. Sargent, Allen J. York, Jeffery D. Molkentin

**Affiliations:** 1 Department of Pediatrics, University of Cincinnati, Cincinnati Children’s Hospital Medical Center, Cincinnati, Ohio, United States of America; 2 Howard Hughes Medical Institute, Cincinnati Children’s Hospital Medical Center, Cincinnati, Ohio, United States of America; Rutgers New Jersey Medical School, UNITED STATES

## Abstract

Nemo-like kinase (NLK) is an evolutionary conserved serine/threonine protein kinase implicated in development, proliferation and apoptosis regulation. Here we identified NLK as a gene product induced in the hearts of mice subjected to pressure overload or myocardial infarction injury, suggesting a potential regulatory role with pathological stimulation to this organ. To examine the potential functional consequences of increased NLK levels, cardiac-specific transgenic mice with inducible expression of this gene product were generated, as well as cardiac-specific *Nlk* gene-deleted mice. NLK transgenic mice demonstrated baseline cardiac hypertrophy, dilation, interstitial fibrosis, apoptosis and progression towards heart failure in response to two surgery-induced cardiac disease models. In contrast, cardiac-specific deletion of *Nlk* from the heart, achieved by crossing a *Nlk-loxP* allele containing mouse with either a mouse containing a β-myosin heavy chain promoter driven Cre transgene or a tamoxifen inducible α-myosin heavy chain promoter containing transgene driving a MerCreMer cDNA, protected the mice from cardiac dysfunction following pathological stimuli. Mechanistically, NLK interacted with multiple proteins including the transcription factor Stat1, which was significantly increased in the hearts of NLK transgenic mice. These results indicate that NLK is a pathological effector in the heart.

## Introduction

The adult mammalian heart hypertrophies when faced with a myriad of pathological insults such as hypertension, ischemic heart disease, viral myocarditis and valvular insufficiency [1]. Numerous studies have shown that cardiac hypertrophy is a temporary and adaptive response that reduces wall stress in an attempt to preserve cardiac function [2]. However, prolonged cardiac hypertrophy is maladaptive and can eventually lead to ventricular dysfunction and heart failure characterized by increased rates of myocyte apoptosis, fibrosis and chamber dilation [2]. Multiple signaling pathways are typically involved in mediating the cardiac hypertrophic response, which are initiated by secreted neuroendocrine factors and their membrane-bound receptors with associated G-proteins. Thereafter, intracellular signaling pathways are mobilized that utilize phosphoinositide 3-kinases (PI3Ks)-AKT, calcineurin/nuclear factor of activated T-cells (NFAT), myocyte enhancer fator-2 (MEF2)/histone deacetylases (HDACs), mitogen-activated protein kinases (MAPKs), and many others [2]. These signaling factors then directly augment biosynthetic effectors in the cytosol as well as regulate the activity of transcription factors to alter gene expression, which collectively leads to the selective alterations in gene expression and increased protein synthesis to promote cardiomyocyte hypertrophy.

Nemo-like kinase (NLK), the murine orthologue of *Drosophila* nemo, is an evolutionally conserved serine/threonine protein kinase that is highly expressed in the adult brain, as well as expressed within diverse tissues during embryonic development [3]. Functional studies in lower organisms such as *Drosophila*, *C*. *elegans* and *X*. *laevis* showed that NLK/Nemo plays an important role in selected developmental processes [4] [5]. In mice, *Nlk* deficiency in one background resulted in lethality within 36 hours of birth with cellular hyperplasia and thickening of the alveoli in the lungs [6]. In other genetic backgrounds *Nlk*^*-/-*^ mice are either embryonic lethal or they survive 4–6 weeks but then die from hematopoietic cell abnormalities with defective bone marrow stroma [7]. NLK also induces apoptotic cell death in multiple mammalian cell types [8, 9].

NLK functional effects involve the direct phosphorylation of downstream transcription factors that either activate or inhibit gene expression. For example, active NLK phosphorylates lymphoid enhancer factor 1 (LEF1) to prevent the transcriptional activity of the Wnt/β-catenin-LEF regulatory circuit [10]. Similarly, NLK was shown to phosphorylate FOXO1, thereby excluding this factor from the nucleus to inhibit target gene expression [11].

Here we determined that NLK is induced in diseased mouse hearts, suggesting a functional effect in this highly differentiated tissue. Mechanistically, overexpression of NLK was a potent pathological effector in the hearts of transgenic mice, while its deletion specifically from myocytes of the heart protected against progression towards heart failure following pathologic stimuli. Using a proteomic approach NLK was shown to bind the transcription factor Stat1, which is dramatically upregulated in NLK transgenic hearts. Together, our study shows that NLK serves as a pathological signaling effector in the heart, in association with alterations in Stat1 expression.

## Materials and Methods

### Animals and generation of cardiac-specific NLK genetically modified mice

To generate inducible cardiac-specific NLK transgenic mice, a mouse cDNA encoding this protein was cloned into *Sal*I and *Hin*dIII sites of the modified murine α-myosin heavy chain (αMHC) promoter expression vector to allow for doxycycline (Dox)-regulated expression of NLK in the presence of a cardiac-specific tetracycline transactivator (tTA)-containing transgene [12]. NLK transgenic mice were genotyped using the following primers: forward, 5’-cagggaagtggtggtgtag-3’, reverse, 5’-accgaattctcacgaggagacggcgg-3’. Dox (625 mg/kg) was administered in the food until weaning [13]. Mice were then switched to a regular chow diet for an additional 5~6 weeks to allow NLK expression as Dox levels in the mice cleared. To generate cardiac-specific *Nlk* gene-deleted mice, embryonic stem (ES) cells with a knock-out first allele of the *Nlk* gene (Nlktm^2a(KOMP)Wtsi^) were purchased from KOMP Repository and used in aggregation with 8-cell embryos to generate chimeric mice. Germline transmitting male chimeras were crossed with Rosa26-FLPe females (the Jackson Laboratory, #9086) to remove the neomycin cassette and to generate a conditional allele with *loxP* sites flanking exon 2 of *Nlk*. To achieve cardiac-specific deletion of *Nlk*, *Nlk-loxP* (*fl*) mice were crossed with tamoxifen-inducible α-myosin heavy chain (αMHC)-MerCreMer transgenic mice or βMHC-Cre transgenic mice. Primary cultures of neonatal myocytes and nonmyocytes were obtained by enzymatic dissociation of 1–2 day-old Harlan Sprague-Dawley rat neonates as described previously [14]. After digestion, cells were plated for 1.5 h on culture dishes to allow nonmyocytes to attach. The cardiomyocytes (containing less than 10% nonmyocytes) were collected through gently rinsing of the dish, and then plated on gelatinized cell culture dishes. Both myocytes and nonmyocytes were cultured in M199 media supplemented with 2% bovine growth serum (Hyclone, SH 30541) and 100 units/ml penicillin/streptomycin (Sigma, P0781).

### Animal husbandry and ethics statement

Mice were housed in standard barrier rack cages supplied with Purina Rodent Chow 5001 with automatic watering dispensers. Cages were observed daily and were regularly changed by certified veterinary technicians at Cincinnati Children’s Hospital Medical Center. Mice were also closely assessed for their wellbeing such as physical activity and food intake on a daily basis. Standard housing conditions and husbandry conform to AAALAC standards and the institutions ongoing certification by this organization, as well as by the standard guidelines from the Office of Laboratory Animal Welfare (http://grants.nih.gov/grants/olaw/animal_use.htm). All animal experimentation was approved by the Office of Research Compliance and Regulatory Affairs and by the Cincinnati Children’s Hospital Institutional Animal Care and Use Committee (Protocol Number: 2E11104). No human subjects were used.

### Echocardiography, animal surgery and histology

Cardiac function and dimensions were measured by echocardiography with a SONOS 5500 instrument (Hewlett-Packard) and a 15-MHz transducer. Left ventricular (LV) fractional shortening percentage (FS%) was calculated using left ventricle internal diameters at the end of systole and diastole (LVIDs and LVIDd, respectively) according to the formula: ([LVIDd-LVIDs]/LVIDd) ×100(%). All surgeries to induce cardiac pathology were performed on 8~12 week-old age and sex-matched mice. Pathologic hypertrophy in mice was induced by transverse aortic constriction (TAC) as previously described [15]. In brief, transverse aortas were exposed through a thoracotomy procedure to permit constriction with 7–0 silk (Ethicon, 768G) around a 27-guage wire that was removed. An additional group of mice for each genotype was subjected to a sham procedure. At the end of surgery, mice were given the pain medicine buprenex at a final concentration of 0.03 mg/ml by veterinary technicians. Mice were then transferred to 30°C chamber and closely monitored to allow overnight recovery. The next day, mice were transferred back to standard housing and again monitored daily by veterinary technicians. After 2 weeks, Doppler echocardiography was performed to ensure equal pressure gradients across the constrictions between the mice, for which the mice were given 2% isoflurane inhaled anesthetic as measurements were taken.

The surgical procedure for ischemia/reperfusion (I/R) or myocardial infarction (MI) was described previously [16]. Briefly, a suture was tied with a slipknot around the left coronary artery, and mice were revived by removal from 2% isoflurane inhaled anesthetic and 60 minutes later the knot was released under 2% isoflurane-induced anesthesia and the heart was reperfused for 24 hours before analysis of area of infarction injury. For injury analysis, hearts were removed and perfused with 2% triphenyltetrazolium chloride in saline and 2% Evan’s blue dye to identify area at risk, infarct area and area of perfusion [17]. Mice were euthanatized by CO_2_ inhalation for 5 minutes followed by cervical dislocation before the hearts were removed. Hearts were fixed overnight in 10% formalin-containing phosphate buffered saline and dehydrated for paraffin embedding. Serial 5-μm heart sections were stained with Masson’s trichrome to detect interstitial fibrosis visualized as blue. TUNEL analysis to infer apoptosis levels in cardiac myocytes was performed using in situ cell death detection kit (Roche, 12156792910) and α-actinin antibody (Sigma, A7732) reactivity staining on paraffin embedded heart tissue from 4 different mice of each genotype. Approximately 14,000 α-actinin positive myocytes were counted for the TUNEL analysis.

### RNA isolation and quantitative PCR

Total RNA was isolated from mouse hearts with the RNeasy Fibrous Tissue Kit (Qiagen, 74704) and quantified with a NANODROP 2000 Spectrophotometer (Thermo Scientific). cDNA was synthesized using the SuperScript III First-Strand Synthesis Kit (Invitrogen, 18080–051). Quantitative PCR was performed using SYBR green dye (Bio-Rad, 172–5274) on a CFX96 real-time PCR detection system (Bio-Rad). The primer sequences for real-time PCR were as follows: ribosomal protein 27, forward: 5’-ggacgctactccggacgcaaag-3’, reverse: 5’-cttcttgcccatg gcagctgtcac-3’; NLK, forward: 5’-cactcgcatcatccgcaac-3’, reverse: 5’-ttcccggaagactcttttgc-3’; atrial natriuretic factor (ANF), forward: 5’-gccctgagtgagcagactg-3’, reverse: 5’-cggaagctgttgcagccta-3’; b-type natriuretic peptide (BNP), forward: 5’-ctgctggagctgataagaga-3’, reverse: 5’-agtcagaaactggagtctcc-3’; β-MHC, forward: 5’-acctaccagacagaggaaga-3’, reverse: 5’-ttgcaaagagtccaggtctgag-3’; collagen 1α1(Col1a1), forward: 5’- aattcggactagacattggccctg-3’, reverse: 5’- ggttgttcgtctgtttccagggtt-3’; collagen 1α2(Col1a2), forward: 5’- ctggtcttgctggcctacat-3’, reverse: 5’-accctgagaaccacgaacac-3’.

### Immunoprecipitation, nuclear fractionation and Western blotting

HEK293 cells transfected with Flag-NLK cDNA containing expression plasmid were lysed into buffer containing 20 mM HEPES pH 7.4, 0.5% Triton X-100, 150 mM NaCl, 1 mM EDTA, and a protease/phosphatase inhibitor cocktail (Thermo Fisher Scientific, 78440). Anti-Flag M2 magnetic beads (Sigma, M8823) were incubated with the protein lysate for 1 hour at room temperature with rotation, which was then washed three times with lysis buffer and eluted with 2x Laemmli buffer. Protein samples were separated by 10% SDS-PAGE and visualized by silver staining (Sigma, PROTSIL1-1KT). Unique proteins were extracted and identified by mass spectrometry through a core facility at the University of Cincinnati. To further confirm protein interactions, HEK293 cells were transfected with a Flag-NLK cDNA containing expression plasmid for 48 hours, lysed and incubated with anti-Flag M2 magnetic beads to immunoprecipitate endogenous proteins that were subsequently detected by Western blotting. In a parallel experiment, isolated neonatal rat cardiac myocytes were infected with a recombinant adenovirus expressing NLK for 24 hours. Cells were then lysed and the protein samples were incubated with either normal rabbit IgG (Santa Cruz biotechnology, sc-2027) or NLK antibody (Millipore, Ab10206) for 4 hours followed by incubation with protein A magnetic beads (Thermo Fisher Scientific, 88845) for 2 hours. After three washes the beads were eluted with 2x Laemmli buffer and subjected to SDS-PAGE.

Nuclear fractionation of heart tissue was performed as previously described [18]. In brief, fresh heart tissue was subject to Dounce homogenization in hypotonic buffer comprised of 250 mM sucrose, 50 mM Tris-HCl pH 7.4, 5 mM MgCl_2_, protease and a phosphatase inhibitor cocktail (Thermo Fisher Scientific). The homogenate was transferred to new tubes and maintained on ice for 30 minutes, then centrifuged at 800 g for 15 minutes. The supernatant was collected and centrifuged for 10 minutes at 11,000 g to collect the remaining supernatant as the cytosolic fraction. The pellet from the initial homogenization containing nuclei and debris was homogenized again and centrifuged at 500 g for 15 minutes to collect the pellet as the nuclear fraction that was then solubilized in buffer containing 20 mM HEPES pH 7.9, 1.5 mM MgCl_2_, 0.5 M NaCl, 0.2 mM EDTA, 1% Triton X-100, protease and phosphatase inhibitor cocktails (Thermo Fisher Scientific). Equal amount of cytosolic and nuclear proteins were loaded for Western blotting.

The following antibodies were used for Western blot detection: NLK (Millipore, Ab10206), Gapdh (Fitzgerald, 10R-G109A), Flag (Cell Signaling Technology, 2368), α-tubulin (Santa Cruz biotechnology, sc-5286), lamin A/C (Cell Signaling Technology, 2032), Stat1 (Cell Signaling Technology, 9172), Stat3 (Cell Signaling Technology, 12640). Western blots were quantified using NIH ImageJ analysis and based on results from at least three experiments.

### Statistical tests

All the results were presented as mean ± SE. Student *t* test was performed to compare means between 2 groups. 1-way ANOVA with a Bonferroni post hoc test was used for comparison of differences across multiple groups. p<0.05 was considered statistically significant.

## Results

### Cardiac-specific overexpression of NLK causes cardiomyopathy

**Fig 1:**
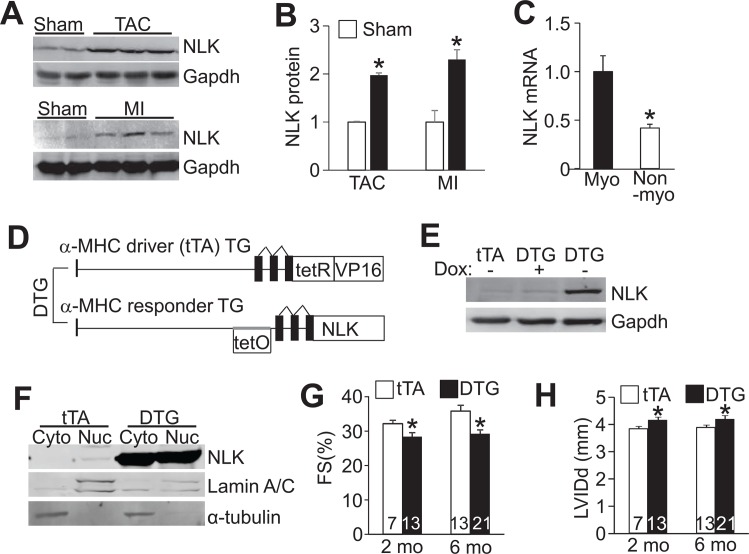
NLK is induced following cardiac stress and its overexpression causes cardiomyopathy in transgenic mice. (A) Western blot analysis of NLK induction in the hearts of mice subjected to 2 weeks of TAC or 1 week after MI. Gapdh was used as a loading control. (B) Quantification of NLK protein induction from experiments as shown in A. *p<0.05 vs sham. (C) Real-time PCR analysis of NLK mRNA expression in myocytes and nonmyocytes isolated from neonatal rat hearts. Myo, myocytes. Nonmyo, non-myocytes. *p<0.05 vs myocytes. (D) Schematic of the double transgenic (DTG) system used to generate cardiac-specific and Dox inducible NLK expressing transgenic mice. (E) Western blot analysis of NLK expression in the hearts of mice fed with Dox containing food during pregnancy and up to weaning (+), or mice 5 weeks after Dox removal and weaning (-). tTA single transgenic control sample is shown for baseline levels of endogenous NLK in the heart. (F) Western blot analysis of NLK distribution in the cytoplasm (cyto) and nucleus (nuc) from either single transgenic tTA control or DTG hearts. Lamin A/C and α-tubulin were used as loading controls for nucleus and cytoplasm, respectively. (G and H) Echocardiographic assessment of left ventricular v(LV) fractional shortening percentage (FS%) and LV internal chamber dimension at diastole (LVIDd) in tTA and DTG mice at 2 and 6 months of age. *p<0.05 vs tTA control. Number of mice analyzed is shown in the bars.

Nemo is a serine/threonine kinase essential for programmed cell death and eye development in *Drosophila* [19, 20]. NLK is the mammalian orthologue of *Drosophila* nemo implicated in multiple physiological processes [21–23]. We suspected that NLK might play a regulatory role in the heart given an observed upregulation of its expression following 2 weeks of pressure overload hypertrophy induced by transverse aortic constriction (TAC) and 1 week after myocardial infarction (MI) injury (Fig 1A and 1B). Myocytes isolated from neonatal rat hearts were the predominant source of NLK mRNA compared with the nonmyocyte fraction (Fig 1C). To model this profile of increased NLK expression in the disease stimulated mouse heart, we generated inducible transgenic mice with cardiac-specific expression of NLK using a modified murine α-myosin heavy chain (α-MHC) promoter-based double-transgenic (DTG) expression system that was tetracycline responsive (Fig 1D). To prevent potential postnatal developmental effects by NLK overexpression, Dox-containing food was administered to the pregnant mothers with continuous feeding for the first 3 weeks after birth until weaning. Under these conditions the DTG mice are repressed for NLK expression when Dox is present (Fig 1E, lane 2), but with Dox removal for 5~6 weeks NLK protein expression is induced (Fig 1E, lane 3). We also performed cellular protein subfractionation for NLK in both tTA control and NLK DTG hearts, which showed that NLK was targeted to both the cytoplasm and nucleus (Fig 1F), consistent with subcellular fractionation data from COS7 cells [3].

**Fig 2:**
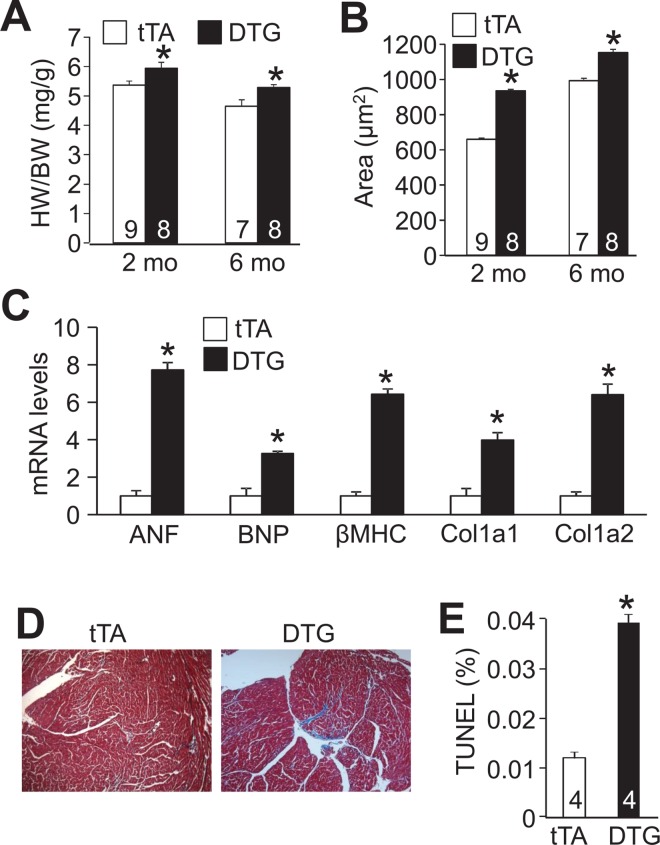
NLK DTG mice develop cardiomyopathy. (A) Heart weight (HW) normalized to body weight (BW) in tTA and NLK DTG mice at the indicated ages. Number of mice used is shown in the bars. *p<0.05 vs tTA. (B) Cross-sectional area of cardiomyocytes from histological heart sections from tTA and DTG mice analyzed by ImageJ software. Number of mice used is shown in the bars. At least 600 cardiomyocytes were analyzed for each group across the number of hearts shown. *p<0.05 vs tTA. (C) Real-time PCR analysis of mRNA for the indicated genes from the hearts of tTA control and DTG mice at 2 months of age. At least 4 hearts were used for each group and all PCR samples were performed in duplicate. *p<0.05 vs tTA. (D) Representative Masson’s trichrome-stained histological heart sections from tTA and NLK DTG mice at 2 months of age. Magnification is 100x total. (E) Immunohistochemical assessment of cardiac myocytes positive for both TUNEL and α-actinin antibody staining in histological sections from hearts of tTA or DTG mice. *p< 0.05 vs tTA (at least 14,000 nuclei were counted across 4 hearts in each group).

Associated with NLK induction in the heart upon Dox removal, NLK DTG mice showed a significant impairment in cardiac fractional shortening (FS) and left ventricular (LV) chamber dilation at both 2 and 6 months of age as assessed by echocardiography (Fig 1G and 1H). NLK DTG mice also had increased heart/body weight ratios as well as an increase in the cross-sectional area of individual cardiomyocytes within their heart, indicating cardiac hypertrophy (Fig 2A and 2B). NLK overexpression also led to increased mRNA levels of cardiac hypertrophic genes atrial natriuretic factor (ANF), b-type natriuretic peptide (BNP) β-MHC and induction of the generalized cardiac fibrosis marker collagen 1a1 and collagen 1a2 and a corresponding increase in Masson’s trichrome staining for fibrosis from histological heart sections (Fig 2C and 2D). Consistent with its previously reported role as a cell death regulator, NLK overexpression in the heart also resulted in a significant increase of TUNEL in cardiac myocytes co-stained with α-actinin (Fig 2E).

### Involvement of NLK in cardiac pathology in vivo

**Fig 3:**
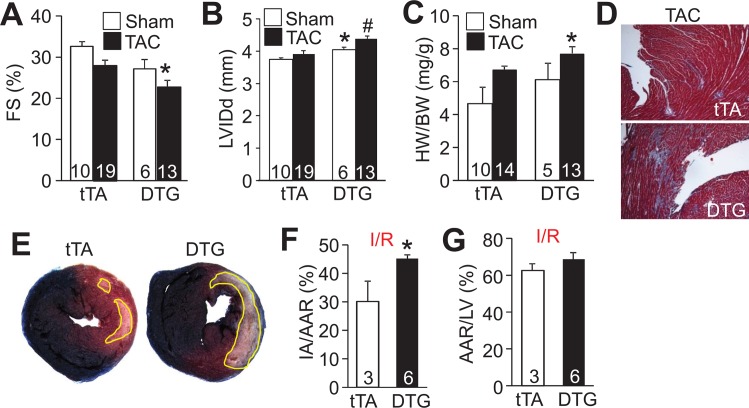
Analysis of cardiac hypertrophy and cell death in NLK DTG after stress stimulation. (A and B) Echocardiographic assessment of FS% and LVIDd in tTA and DTG mice after 2 weeks of a sham or TAC surgical procedure. Number of mice analyzed is shown in the bars. *p<0.05 vs sham; #p<0.05 versus tTA TAC. (C) HW normalized to BW in tTA and NLK DTG mice 2 weeks after sham or a TAC surgical procedure. Number of mice used is shown in the bars. *p<0.05 vs tTA TAC. (D) Representative Masson’s trichrome-stained histological heart sections from tTA and NLK DTG mice after 2 weeks of TAC. Magnification is 100x total. (E) Representative images of transverse heart sections stained with triphenyltetrazolium chloride (red area) following I/R injury. Ischemic area is outlined in yellow. (F) Quantification of the ischemic area versus the area at risk (IA/AAR) for the indicated groups of mice. *p<0.05 vs tTA. Number of mice used is shown in the bars. (G) Quantification of the percent area at risk (AAR) normalized to the left ventricle (LV) area from hearts of tTA or DTG mice after I/R injury. Number of animals used is shown in the bars.

Given the baseline pathology observed in DTG hearts with NLK overexpression, we further analyzed the cardiac stress response of these mice. First, we subjected 8 week-old tTA and DTG mice to a TAC surgical procedure for 2 weeks (5 weeks after Dox removal). Consistent with their previous phenotype, NLK DTG showed even worse ventricular FS and LV chamber dilation after 2 weeks of TAC stimulation compared with tTA controls (Fig 3A and 3B). Heart weight to body weight ratios and interstitial fibrosis were also significantly increased in DTG mice after TAC compared to controls (Fig 3C and 3D), further supporting the hypothesis that NLK is a pathological signaling effector in the heart. We also analyzed cardiomyocyte viability with NLK overexpression in response to 60 minutes of ischemia followed by 24 hours of reperfusion (I/R), a surgery model known to induce tissue damage through cell death. Hearts from NLK DTG mice showed significantly larger infarction areas when normalized to the total area at risk versus tTA control mice, again supporting the pathological role of NLK activity in the heart (Fig 3E–3G).

### Deletion of *Nlk* protects the heart from pathological stimulation

**Fig 4:**
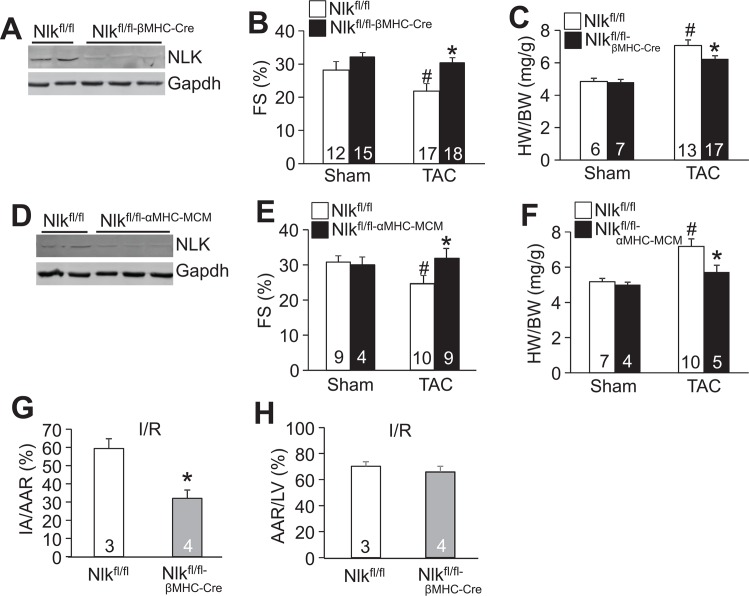
Cardiac-specific deletion of *Nlk* protects mice from stress stimulation. (A) Western blot assessment of NLK cardiac protein levels in 8 week-old *Nlk*^*fl/fl-βMHC-Cre*^ mice versus control *Nlk*^*fl/fl*^ mice. Gapdh was used as a processing and Western blot loading control. (B) LV FS (%) in hearts of the mice shown at 10 weeks of age as either sham operated or 2 weeks after a TAC surgical procedure. Number of mice used is shown in the bars. #p<0.05 versus sham; *p<0.05 versus *Nlk*^*fl/fl*^ TAC. (C) HW/BW ratio in the groups of mice shown at 10 weeks of age as either sham operated or 2 weeks after a TAC surgical procedure. Number of mice used is shown in the bars. #p<0.05 versus sham; *p<0.05 versus *Nlk*^*fl/fl*^ TAC. (D) Western blot assessment of NLK cardiac protein levels in 8 week-old *Nlk*^*fl/fl-αMHC-MCM*^ mice 2 weeks after TAC. Gapdh was used as a processing and Western blot loading control. Mice were given tamoxifen injection via intraperitoneal (IP) injection for 7 consecutive days (0.5 mg/day), then subjected to TAC. (E) LV FS (%) in hearts of the mice shown at 10 weeks of age as either sham-operated or 2 weeks after a TAC surgical procedure. Number of mice used is shown in the bars. #p<0.05 versus sham; *p<0.05 versus *Nlk*^*fl/fl*^ TAC. (F) HW/BW ratio of the groups of mice shown at 10 weeks of age as either sham operated or 2 weeks after a TAC surgical procedure. Number of mice used is shown in the bars. #p<0.05 versus sham; *p<0.05 versus *Nlk*^*fl/fl*^ TAC. (G) Quantification of the ischemic area versus the area at risk (IA/AAR) for the indicated groups of mice. *p<0.05 vs *Nlk*^*fl/fl*^. Number of animals used is shown in the bars. (H) Quantification of the area at risk (AAR) to the total LV area from the hearts of mice show in G. Number of animals used is shown in the bars.

While overexpression of NLK suggested a pathologic role in the heart, it is possible that the very nature of the overexpression approach itself disrupts the true physiologic function of this protein. Hence a potentially more powerful approach towards understanding the biologic function of NLK in the heart is by deleting this gene, which we achieved here by creating *Nlk* gene-targeted mice. However, because mice deficient in *Nlk* are embryonic, perinatal or early juvenile lethal depending on the strain background, here we created *Nlk-loxP (fl)* targeted mice to enable tissue-specific and regulated deletion of this gene in conjunction with a Cre-expressing allele. *Nlk*^*fl/fl*^ mice were first crossed with a β-MHC-Cre transgenic line for cardiac deletion of this gene in the developing embryonic heart and thereafter, which when assayed as young adult mice showed a ~90% loss of total protein from the heart (Fig 4A). These mice were then subjected to 2 weeks of TAC stimulation to induce cardiac hypertrophy and heart failure. *Nlk*^*fl/fl-βMHC-Cre*^ mice deficient in NLK protein showed significantly less cardiac hypertrophy and a resistance against heart failure as suggested by a reduction in FS compared with *Nlk*^*fl/fl*^ control mice subjected to 2 weeks of TAC (Fig 4B and 4C). This entire approach was repeated in separate groups of *Nlk*^*fl/fl*^ mice, but instead crossed with tamoxifen-inducible α-MHC-MerCreMer (MCM) transgenic mice for adult-specific deletion of *Nlk*. Due to low expression of NLK at baseline in adult hearts, the efficiency of NLK deletion with the α-MHC-MCM transgene was examined with 7 days of tamoxifen administration in TAC operated mice, which showed an ~80% reduction of protein (Fig 4D). Adult-specific deletion of *Nlk* also significantly protected mice from cardiac hypertrophy and a loss of ventricular performance 2 weeks after TAC stimulation, versus *Nlk*^*fl/fl*^ control mice (Fig 4E and 4F). Finally, *Nlk* heart-specific deleted mice also showed smaller infarct areas compared with control mice following I/R injury, whereas the total area at risk between the two groups was similar (Fig 4G and 4H). Taken together, deletion of *Nlk* protects the mouse heart from pathology associated with pressure overload and infarction injury, strongly suggesting that this kinase is normally a maladaptive signaling effector in the heart.

### Stat1 protein levels are altered in NLK DTG hearts

**Fig 5:**
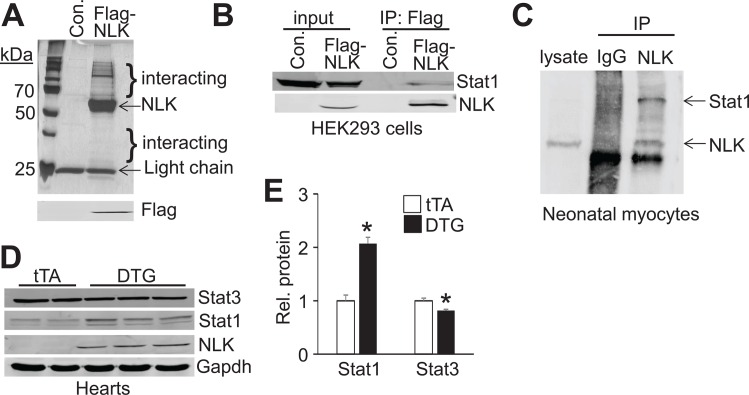
NLK interacts with Stat1. (A) Top, silver stained 10% SDS-PAGE gel of immunoprecipitated protein samples from HEK293 cells transfected with either an empty Flag containing expression plasmid or Flag-NLK expression plasmid. Bottom, Western blot for Flag-tagged NLK protein eluted from anti-Flag M2 magnetic beads. Two pools of interacting proteins were extracted and analyzed by mass spectrometry. IgG light chain protein is non-specific but shows the same loading of anti-Flag M2 beads in the 2 samples. (B) Western blot validation of the interaction between overexpressed NLK and endogenous Stat1 from HEK293 cells. (C) Immunoprecipitation followed by Western blotting to show that overexpressed NLK interacted with endogenous Stat1 in isolated neonatal cardiac myocytes. (D) Western blot analysis for Stat3, Stat1, and NLK in tTA and DTG hearts at 3 months of age with ~9 weeks of NLK induction after Dox removal. Gapdh was used as a processing and loading control. (E) Quantification of expression levels of Stat1 and Stat3 shown in D. These data were generated from at least two experiments, 3 mice each. *p<0.05 vs tTA.

To begin to investigate potential downstream mechanisms whereby NLK signaling might mediate pathological effects when induced in the heart, we instituted a proteomic approach to identify potential interacting partners. For sensitivity reasons we had to resort to NLK overexpression by transfection in HEK293 cells using a Flag-tagged NLK cDNA containing expression plasmid, followed by immunoprecipitation with anti-Flag M2 antibody-conjugated magnetic beads. Protein eluates from these beads were subjected to SDS-PAGE and silver staining followed by mass spectrometry to potentially identify novel interacting proteins (Fig 5A). The control was an empty Flag expressing plasmid processed under identical conditions (Fig 5A). A number of specific proteins were identified as NLK interacting factors, of which Stat1 was considered interesting and subjected to further analysis. Stat1 is a transcription factor typically activated by Janus tyrosine kinases (JAK) during inflammatory or other signaling responses [24, 25]. We further validated the specificity of this interaction in separate transfection and immunoprecipitation assays from HEK293 cells, which again showed interaction of NLK with endogenous Stat1 (Fig 5B). To determine if Stat1 might also be an effector in the heart downstream of NLK signaling, we confirmed their interaction in isolated neonatal rat cardiomyocytes by immunoprecipitation of overexpressed NLK to detect endogenous Stat1 with subsequent western blotting (Fig 5C). To study whether Stat1 levels were altered upon NLK overexpression in the heart, we performed Western blotting from NLK DTG hearts, which showed a remarkable upregulation of total Stat1 protein and a slight but significant down regulation of Stat3 (Fig 5D and 5E). Stat1 was previously shown to be a pathological effector in the heart, as loss of this gene protected the heart after ischemia injury [26]. In contrast, Stat3 was previously shown to be a cardioprotective factor as deletion of this gene promoted inflammation, fibrosis and heart failure [27]. These data suggest that the pathological phenotype observed in NLK-DTG mice is associated with increased expression of Stat1 in the heart, which given its ability to directly interact might be part of the pathologic mechanism.

## Discussion

NLK has been extensively studied given its important role in developmental processes, cell survival and proliferation. Prior to this report nothing was known of NLK's activity or functional effects in the heart, despite the fact that this kinase is highly induced with pathologic stimulation. Here we determined that mice with inducible and cardiac-specific expression of NLK develop baseline cardiomyopathy and are more susceptible to heart failure upon pressure overload, as well as to greater myocyte death with I/R injury. More importantly, deletion of *Nlk* specifically from the heart, either during embryonic development or for the first time in adults, reduced the hypertrophic response to pressure overload stimulation and lessened propensity towards heart failure and reduced cardiac functional capacity. Finally, heart-specific deletion of *Nlk* significantly protected the heart from I/R injury, consistent with its previous description as a regulator of apoptosis in cultured cells [9, 23]. Taken together, these observations indicate that NLK is a maladaptive signaling kinase in the heart that mediates disease, hence it represents a novel target for potential pharmacologic intervention in treating human heart disease, and as such, selective kinase inhibitors should be developed.

NLK is most related to the extracellular signal-regulated kinases1/2 (ERK1/2) of the MAPKs, and like ERK1/2 is activated by a diverse array of membrane-based signals [3, 22]. While NLK has to become phosphorylated for its activity, it is less clear how this is directly achieved, although TGF-β-activated kinase 1 (TAK1) has been implicated as an upstream regulator, and NLK is known to autophosphorylate itself and become active when overexpressed, presumably suggesting that its transcriptional induction alone might lead to greater activity [5, 28]. Once activated, NLK has been reported to directly phosphorylate and regulate the activity of the transcription factors LEF1, FOXO1 and c-Myb [11, 29, 30].

To understand how NLK might be having a pathologic effect in the heart, we screened for NLK interacting factors using a proteomic approach, which identified Stat1, a transcription factor that was previously shown to play a pathologic signaling role in the heart [26, 31]. Stat1 transcriptional activity is induced by cytokine and growth factor signals that induce phosphorylation and activation of the JAKs and MAPKs [32]. In the current study, we observed elevated levels of Stat1 and a subtle but significant decrease of Stat3 in the hearts of NLK DTG mice. These observations are consistent with the opposing actions of Stat1 and Stat3 on apoptotic cell death in various cell types [33, 34]. For example, overexpression of Stat1 in cultured cardiomyocytes enhances apoptotic cell death, while inhibition of Stat1 by antisense oligonucleotides decreased cell death in response to I/R injury [35]. However, the dependence of Stat1 on apoptosis dependent gene expression requires its phosphorylation at serine 727, which was not detected here using either a ser727-Stat1 antibody or by Phos-Tag PAGE, suggesting NLK might function through another mechanism if it is truly a causal regulator downstream of NLK (data not shown). In the future it will be interesting to determine if loss of Stat1 in genetically modified mice could rescue part of the cardiac pathology associated with NLK overexpression.
